# One-Step Recovery of scFv Clones from High-Throughput Sequencing-Based Screening of Phage Display Libraries Challenged to Cells Expressing Native Claudin-1

**DOI:** 10.1155/2015/703213

**Published:** 2015-11-15

**Authors:** Emanuele Sasso, Rolando Paciello, Francesco D'Auria, Gennaro Riccio, Guendalina Froechlich, Riccardo Cortese, Alfredo Nicosia, Claudia De Lorenzo, Nicola Zambrano

**Affiliations:** ^1^Dipartimento di Medicina Molecolare e Biotecnologie Mediche, Università degli Studi di Napoli Federico II, Via S. Pansini 5, 80131 Napoli, Italy; ^2^CEINGE Biotecnologie Avanzate S.C. a R.L., Via G. Salvatore 486, 80145 Napoli, Italy; ^3^Associazione Culturale DiSciMuS RFC, 80026 Casoria, Italy

## Abstract

Expanding the availability of monoclonal antibodies interfering with hepatitis C virus infection of hepatocytes is an active field of investigation within medical biotechnologies, to prevent graft reinfection in patients subjected to liver transplantation and to overcome resistances elicited by novel antiviral drugs. In this paper, we describe a complete pipeline for screening of phage display libraries of human scFvs against native Claudin-1, a tight-junction protein involved in hepatitis C virus infection, expressed on the cell surface of human hepatocytes. To this aim, we implemented a high-throughput sequencing approach for library screening, followed by a simple and effective strategy to recover active binder clones from enriched sublibraries. The recovered clones were successfully converted to active immunoglobulins, thus demonstrating the effectiveness of the whole procedure. This novel approach can guarantee rapid and cheap isolation of antibodies for virtually any native antigen involved in human diseases, for therapeutic and/or diagnostic applications.

## 1. Introduction

Monoclonal antibodies (mAbs) represent valuable tools in biological treatments for a variety of clinical conditions, including viral infections and cancer. Screening of antibody libraries by phage display allows for rapid selection of single-chain variable fragments (scFvs), from which to isolate the sequences of variable heavy (VH) and variable light (VL) chains for mAb conversion. Thus, avoiding animal immunization, it is possible to obtain antibodies against toxic or highly conserved antigens, or against plasma membrane proteins or receptors, in their native conformation [1, 2]. This possibility is of relevance, for isolation of antibodies, to interfere with viral infections. In the paradigm of viral hepatitis, mAbs have been generated, preventing hepatitis C virus (HCV) infection of hepatocytes. HCV utilizes a set of different cell membrane receptors to infect liver cells: CD81, SR-BI, and the tight junction proteins CLDN1 and OCLN [1, 3–6]. CD81 and SR-BI mAbs actually inhibit HCV infection, both* in vitro* and* in vivo* [7]. Non-human or chimeric anti-CLDN1 antibodies were shown to be effective against HCV infection* in vitro* and* in vivo* [8–11]. So far, no fully human anti-CLDN1 or OCLN mAbs are available. Still, generation of novel mAbs is a relevant issue, even though antiviral drugs, such as boceprevir and telaprevir, are currently in clinical use. However, besides their toxic side effects, their use may be limited by the occurrence of drug-resistant phenotypes [12–16]. Furthermore, these antiviral drugs are not as effective to prevent graft reinfection in patients subjected to liver transplantation, since the treatment is delayed until several months from surgery [17].

High-throughput sequencing (HTS) was successfully applied to phage display technology, to get full advantage from screening of phage display libraries [18, 19]. It allows us to rapidly identify the potential binders of a given antigen, based on the counts of the corresponding scFv fragments, within a cycle, and on the kinetic of their enrichments, within consecutive cycles; that may provide useful information on the whole screening. After their identification, the clones of interest need to be recovered from the DNA library of the relevant selection cycle, for validation of binding. HTS-based selection of phage display libraries should provide rapid information on the screening progression and a comprehensive set of scFv clones, since it limits the possibility to loose potential good binders during the repetitive handling of clones, which is required during a classical screening. The bottleneck of a HTS-based screening is, however, the recovery of scFv clones of interest. The availability of a set of alternative strategies, to recover rapidly the clones of interest, would allow us to overcome the limiting step in HTS-based screening of phage display libraries [19]. In this paper, we tested the whole procedure of a HTS-based screening, to isolate binders of native CLDN1 protein, expressed on the cell surface of mammalian cells. We successfully identified a set of 75 potential binders of CLDN1, from which novel, human antibodies could be isolated, possessing the ability to interfere with HCV infection. We also implemented a rapid and effective method, for one-step recovery of scFv clones from the enriched population of fragments. This method was applied to some scFv fragments, characterized by heavy-chain complementarity determining regions 3 (HCDR3) of different length, to demonstrate its effectiveness in the generation of complete and functional monoclonal antibodies.

## 2. Materials and Methods

### 2.1. Cell Cultures

The Human Embryonic Kidney HEK 293T cells were cultured in standard conditions using Dulbecco's Modified Eagle's medium (DMEM, Life Technologies, Inc., Paisley, UK) with the addition of nonessential amino acid solution (Gibco, Life Technologies, Inc.). The HEK 293T cells transduced with the gene encoding CLDN1 [1] were grown in DMEM containing Blasticidin (2 *μ*g/mL) (Gibco, Life Technologies, Inc.). Media were supplemented with 10% FBS, 50 units/mL penicillin, and 50 *μ*g/mL streptomycin (all from Gibco, Life Technologies, Inc.).

### 2.2. Selection of scFv Phage on Living Cells

The phage library was grown in 2xTY medium containing 100 *μ*g/mL of Ampicillin and 1% glucose up to an optical density at 600 nm (OD600) of 0.5. Subsequently, 1 × 10^9^ plaque-forming units of M13-K07 helper phage encoding trypsin-cleavable pIII protein were added to 25 mL of culture and were grown for 1 hour. The bacterial cells were then pelleted through centrifugation for 15 minutes at 4,000 rpm and then resuspended and grown overnight in 500 mL of 2xTY containing 100 *μ*g/mL of Ampicillin and 25 *μ*g/mL of Kanamycin at 30°C. Phages were collected by two steps of precipitation with polyethylene glycol (PEG) and resuspended in PBS. The theoretical diversity of naïve library was about 1 × 10^10^.

Both HEK 293T cells, mock and transduced with CLDN1 cDNA, were detached by using cell dissociation solution (Sigma-Aldrich, Saint Louis, USA) and washed with PBS. Phages (10^13^ pfu) were blocked with 5% milk powder (Sigma-Aldrich) in PBS for 15 minutes and submitted to two rounds of negative selection by incubation with HEK 293T mock cells (5 × 10^6^) for 2 hours at 4°C. The unbound phages were recovered from supernatant after centrifugation at 1,200 rpm for 10 minutes and then were used for the positive selection performed on CLDN-1 transduced HEK 293T (1 × 10^6^), by incubation for 16 hours at 4°C. Cells were recovered by centrifugation at 1,200 rpm for 10 minutes and washed twice with PBS. Bound phages from each selection were eluted from CLDN-1 transduced HEK 293T with a solution of 1 *μ*g/mL of Trypsin (Sigma-Aldrich), which was then inhibited by EDTA-free protease inhibitor cocktail (Roche Diagnostic, Mannheim, Germany). The recovered phages were amplified by infecting* E. coli* TG1 cells to prepare phage for the following round of selection. Four whole cycles of selection were performed.

### 2.3. VH Extraction and Purification

**Figure 1:**
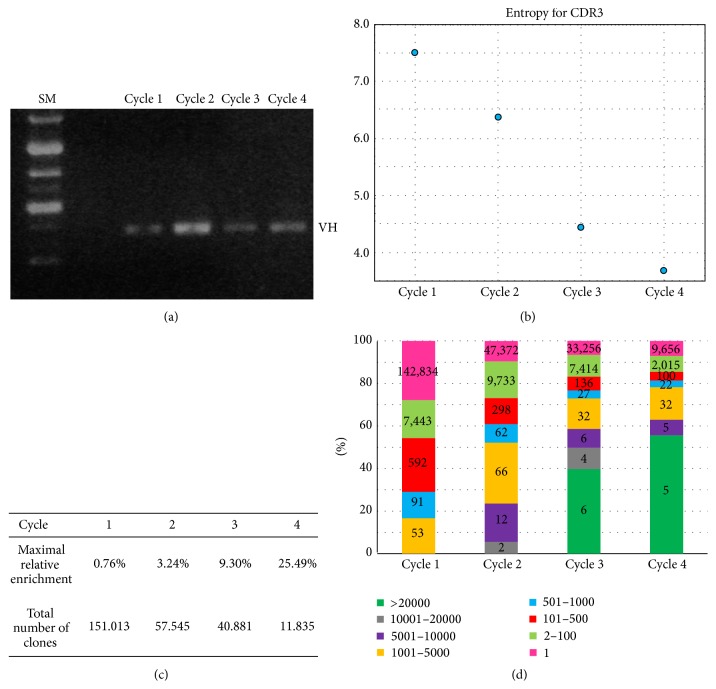
Library screening and analysis of sequences. (a) The panel shows the DNA fragments, gel-purified from sublibraries, after the indicated selection cycles. The corresponding plasmid preparations were digested with* Nco*I and* Xho*I restriction endonucleases, to release the DNA fragments encoding for the VH regions of the scFv fragments. The fragments were bar-coded and subjected to high-throughput sequencing, as described in the text. SM, size marker. (b) The chart reports the entropy values for the populations of fragments originating from the indicated selection cycles, after sequencing. (c) The reported values indicate the total number of clones, and the relative representation of the most abundant clone, within the corresponding selection cycles. (d) The chart indicates the relative distribution of clones, according to the number of counts observed, within the indicated ranges, for each of the 4 selection cycles. Cycles 3 and 4 show similar distributions.

The double strand DNA plasmids containing the scFvs were isolated from each cycle of selection from a culture of superinfected* E. coli* TG1 cells using GenElute HP Plasmid Maxiprep Kit (Sigma-Aldrich). The VHs were excised by double digestion with restriction enzymes* Nco*I and* Xho*I (New England Biolabs) and then purified from a 1.2% agarose gel (Figure 1(a)).

### 2.4. High-Throughput Sequencing

Library preparations of the fragments, sequencing reactions, and preliminary analysis of the data were performed at the Center for Translational Genomics and Bioinformatics, Hospital San Raffaele, Milano, Italy. Briefly, for the preparation of the bar-coded libraries, TruSeq ChIP sample prep kit (Illumina) was used on the VH DNA samples isolated from cycles 1–4. A complementary scheme for bar-coding was implemented, in order to perform sequencing reactions from mixtures of subcycles 1 and 4 (run #1) and of subcycles 2 and 3 (run #2). The bar-coded samples were diluted to a final concentration of 10 pM and sequenced with 2 × 300 nt SBS kit v3 on an Illumina MiSeq apparatus.

### 2.5. scFv Recovery from the Enriched Sublibrary

The three selected clones were isolated from the population of scFv at cycle 3. The QuickChange II XL Site-Directed Mutagenesis Kit (Agilent Technologies) was used to perform extension reactions with overlapping primers, designed within the corresponding HCDR3 regions.

The extension reactions were assembled as follows: 50–250 ng of template; 2/5 *µ*L QuickSolution reagent; 1 *µ*L* Pfu* Ultra High Fidelity DNA polymerase (2.5 U/*µ*L); 5 *µ*L 10x reaction buffer; 1 *µ*L dNTP mix; 125 ng forward primer; 125 ng reverse primer; H_2_O to a final volume of 50 *µ*L.

The primers used were

 3_2 forward 5′-GAGTTATTATCCATTTGACTACT-3′; 3_2 reverse 5′-AGTAGTCAAATGGATAATAACTC-3′; 3_5 forward 5′-CGAGAGACTACTACGGACTTGACTACTG-3′ 3_5 reverse 5′-CAGTAGTCAAGTCCGTAGTAGTCTCTCG-3′; 3_67 forward 5′-CGCGTGGGGCAGGAGGAGCCTTTGACTACTG-3′; 3_67 reverse 5′-CAGTAGTCAAAGGCTCCTCCTGCCCCACGCG-3′.

The template DNA was removed by restriction with 1 *µ*L of* Dpn*I enzyme, as suggested by the kit provider. An appropriate amount of reaction was used to transform XL10-GOLD ULTRACOMPETENT CELLS (Agilent Technologies) and then plated on LB/agar containing 100 *µ*g/mL Ampicillin. Some colonies were picked and the screen success was evaluated by double digestion and sequencing.

### 2.6. Preparation of Phage Particles

Electrocompetent TG1 cells were transformed with dsDNA plasmid of rescued clones and grown in 100 *µ*L of 2xTY medium containing 1% glucose, 25 *μ*g/mL Kanamycin, and 100 *µ*g/mL Ampicillin for 18 hours at 37°C. Then TG1 cells were infected with the M13-K07 helper phage. The culture was centrifuged at 1200 rpm for 30 min to pellet bacteria and recover the scFv phage containing supernatant useful for ELISA. PEG precipitation was used as previously described to concentrate phage particles.

### 2.7. Antibody Production and Purification

For the conversion of the selected scFvs into whole IgG4, the VHs and VLs were amplified by PCR and purified by agarose gel. Then, In-Fusion HD cloning kit (Clontech Laboratories, Mountain View, CA, USA) was used to insert the variable fragments in vectors expressing the constant antibody heavy and light chains. The VHs were cloned in the linearized (*Bam*HI/*Bss*HII) Peu 8.2 vector and the VLs were cloned in linearized (*Apa*LI/*Avr*II) Peu 4.2 vector. Stellar Competent Cells (Clontech Laboratories, Inc., Mountain View, CA, USA) were transformed with obtained vectors, and the colonies were screened by digestion and sequence analysis. The correct preps were cotransfected in HEK293-EBNA by using Lipofectamine Transfection Reagent (Life Technologies, Inc.) and grown up for about 10 days at 37°C in serum-free CD CHO medium (Gibco, Life Technologies, Inc.) in 6-well plates. The conditioned media were collected and the antibodies were purified by using Protein A HP SpinTrap (GE Healthcare Life Sciences, New York, USA). The primers used were the following:

*For VH*

 3_2; 3_5; 3_67: 5′-CTCTCCACAGGCGCGCACTCCGAGGTGCAGCTGTTGGAGT;

*Rev_VH*

 3_2; 3_5 5′-GGCATTGGGTGGATCCGTCGACAGGACTCACCACTCGAGACGGTGACCATTGTCCC;
 3_67 5′-GGCATTGGGTGGATCCGTCGACAGGACTCACCACTCGAGACGGTGACCGTGGTCCC;

*For_VL*

 3_67 5′-CTCCACAGGCGTGCACTCCCAGTCTGTGTTGACGCAGCCG;
 3_2 5′-CTCCACAGGCGTGCACTCCCTTAATTTTATGCAGACTCAGCCCC;
 3_5 5′-CTCCACAGGCGTGCACTCCCAATCTGCCCTGACTCAGCCT;

*Rev_VL*

 3_2 3_5 3_67 5′-TTCTGACTCACCTAGGACGGTCAGCTTGGTCCCTCC.

### 2.8. ELISA 

To confirm the binding specificity for CLDN1 of the selected scFv phages or purified mAbs, cell ELISA were performed by using HEK293 T CLDN-1 positive and mock cells. The cells were detached with nonenzymatic cell dissociation solution (Sigma-Aldrich) and washed with PBS and then resuspended in PBS/BSA 6% in 96 multiwell plates (2 × 10^5^cells/well). The phages or mAbs were added to plate and incubated for 30 minutes at RT. The following antibodies were used to reveal binding of phage-scFvs or of the corresponding antibodies: mouse HRP-conjugated anti-M13 mAb (GE Healthcare Bio-Sciences AB, Uppsala, Sweden); goat HRP-conjugated anti-human IgG (Promega Corporation Madison, USA). After 3 washes cells were resuspended and incubated for 2 minutes in 50 *µ*L of TMB reagent (Sigma-Aldrich). After the incubation, the reaction was stopped through addition of 50 *µ*L of 1 N HCl, and the absorbance (A450) was measured.

## 3. Results 

### 3.1. HTS-Based Screening of a Phage Display Library on CLDN1 Expressing Cells

For isolation of CLDN1 scFvs, the phage display library was subjected to 4 selection cycles; each cycle consisted of a subtractive step on HEK-293 cells, not expressing the antigen on the cell membrane, followed by panning on HEK-293 cells transduced with CLDN1 construct [1]. In order to maximize the exposure of proteins on the cell membrane, panning and the subtractive steps were performed on suspension cultures. Phages from each selection step were collected and amplified for recovery of dsDNA phagemid. DNA preparations were digested with* Nco*I and* Xho*I restriction endonucleases to excise the subcollections of VH fragments (Figure 1(a)). The isolation of the VH fragments (350 bp on average) was preferred to the isolation of the whole scFv fragments (about 750 bp in length), in order to get full sequencing coverage of the most variable HCDR1, HCDR2, and HCDR3 regions. In order to minimize loss of representation of clones, we preferred excision of the VH fragments by restriction enzyme digestion, rather than their amplification by PCR. Thus, the unique amplification step of the whole procedure was implemented for bar-coding of the sublibraries. The bar-coded VH fragments from the four selection cycles were finally sequenced on a MiSeq Illumina platform (see Section 2). We also combined cycles 1 and 4 in a run and cycles 2 and 3 in an additional run to test the possibility to further reduce the costs of the analyses. The aim of analysis was to reveal the most abundant clones, as well as their enrichment profiles throughout the selection rounds.

As a parameter of complexity of 4 sublibraries, we initially explored the number and the diversity of HCDR3s from each selection cycle, through evaluation of the entropy (Figure 1(b)); a strong decrease of entropy occurred throughout the 4 cycles of selection. Accordingly, the relative representation of the most abundant clone inside each sublibrary was progressively increasing over cycles (maximal relative representation from 0.76% to 25.49%), while the complexity (i.e., the number of different clones) was accordingly decreasing over more than one order of magnitude (Figure 1(c)). Finally, as detailed in Figure 1(d) during the selection cycles, we observed that an increasing percentage of sublibraries was occupied by VH fragments with high counts, until cycle 3; cycle 4 showed distributions of counts, similar to those observed in cycle 3, thus indicating that selection of CLDN1 binders was* bona fide* completed after three cycles.

### 3.2. Recovery of scFv Clones from Sublibraries

As shown in Figure 1(d), cycles 3 and 4 show similar distributions of clones characterized by high counts. For further analysis, we focused on clones, for which relative representation was above 1 × 10^−3^. Cycle 3 gave the highest number of clones above such threshold, 75, versus 63 clones from cycle 4.

**Figure 2:**
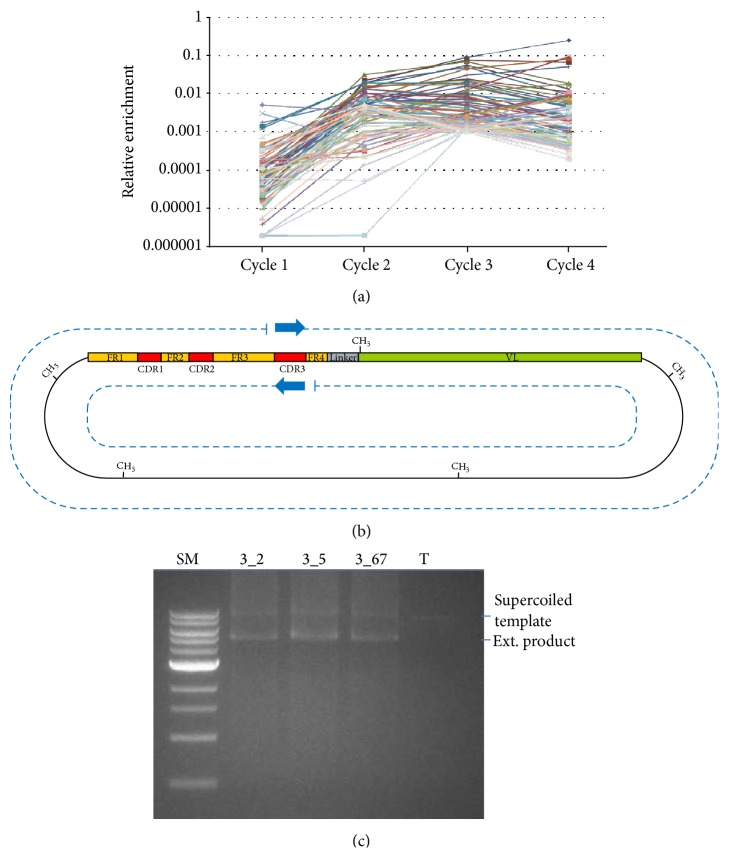
Selection of scFv clones and strategy for recovery. (a) The chart reports the relative enrichments, within the indicated selection cycles, for 75 scFv clones. The threshold for inclusion was arbitrary set to a relative representation value of 1 × 10^−3^ (0.1%). Most clones were maximally enriched at cycle 3. Compared to cycle 4, cycle 3 also showed the highest number of different clones with a relative representation >1 × 10^−3^ (75* versus* 63). Cycle 3 was accordingly selected for recovery of scFv clones. (b) The cartoon describes the strategy, implemented for recovery of scFv clones. The methylated template DNA from cycle 3 sublibrary was copied by* Pfu* DNA polymerase from overlapping primers (block arrows), corresponding to specific sequences within HCDR3 region of VH. The dashed lines represent the newly synthesized DNA, nonmethylated, since it was generated* in vitro*. After* Dpn*I digestion, methylated and hemimethylated DNAs are removed, so that the nicked DNA originating from template copy is able to transform competent* E. coli* cells. The originating colonies thus represent the recovered,* bona fide* scFv clones. (c) The panel shows the products of the extension reactions, carried out on template from selection cycle 3, with overlapping primers for HCDR3 regions of clones 3_2, 3_5, and 3_67. The upper bands correspond to the supercoiled, methylated template; the lower bands represent the primer-extended, nicked products. SM, size marker; T, template DNA.

Thus, we analyzed the enrichment profiles for each of the 75 clones from selection cycle 3; as shown in Figure 2(a), most clones were already enriched from cycle 1 to cycle 2; some clones (30 in number) reached their maximal enrichment at cycle 2, while 37 clones were still increasing their representation over cycle 3. The remaining clones (8 in number) showed comparable enrichment values from cycle 2 to cycle 3. Considering cycles 3 and 4, 19 clones were showing increasing enrichments, while 49 actually showed decreased representation at cycle 4. The remaining clones (7 in number) did not show relevant changes from cycle 3 to cycle 4. Thus, most scFv clones reach the maximal enrichment at cycle 3.

We then selected cycle 3 for recovery of the scFv clones. To this aim, we took advantage of an approach, which is routinely used in molecular biology labs, for site-directed mutagenesis (Figure 2(b)). The system allows us to obtain nicked plasmid DNA by enzymatic copy of a template; the* in vitro* generated DNA is then suitable for* E. coli* transformation and isolation of the clones of interest. Thus, we subjected DNA templates from cycle 3 to enzymatic copy with pairs of overlapping primers. The oligonucleotide sequences were designed inside the HCDR3 regions, since they represent the most variable (thus, selective, in terms of DNA sequence) regions in the antibody repertoire. The range in HCDR3 lengths for the 75 clones was from 10 to 24 amino acids. We selected the scFvs of 3 different VH fragments, characterized by CDR3 regions of different lengths: the shortest (10 a.a., clone 3_5) was selected, since it provides the tightest constraint in the design of specific primers. The additional CDR3s were 13 a.a.- (clone 3_67) or 17 a.a.-long (clone 3_2). Clones 3_2 and 3_5 were highly enriched within cycle 3 (corresponding frequencies were, resp., 8 × 10^−2^ and 5 × 10^−2^), while clone 3_67 was close to the lowest enrichment (frequence was 1 × 10^−3^), among the 75 selected.

As shown in Figure 2(c), nicked DNA was generated for each of the selected clones. In order to remove the library template, which could give rise to undesired scFv clones, the samples were digested with* Dpn*I, which cleaves the methylated and hemimethylated templates, while preserving the fully* in vitro* generated nonmethylated DNA. The resulting DNAs were transformed in* E. coli*, to obtain* bona fide* phagemid DNAs corresponding to the selected VHs. The corresponding constructs were isolated from the transformation reactions with occasional retrieval of undesired constructs. Sanger sequencing of the recovered 3_2, 3_5, and 3_67 clones confirmed 100% identity of the VH regions to the HTS data for each of the three clones. Sanger analysis also allowed us to identify their corresponding VL sequences.

### 3.3. Validation of Binding for scFv Fragments and Converted Antibodies

**Figure 3:**
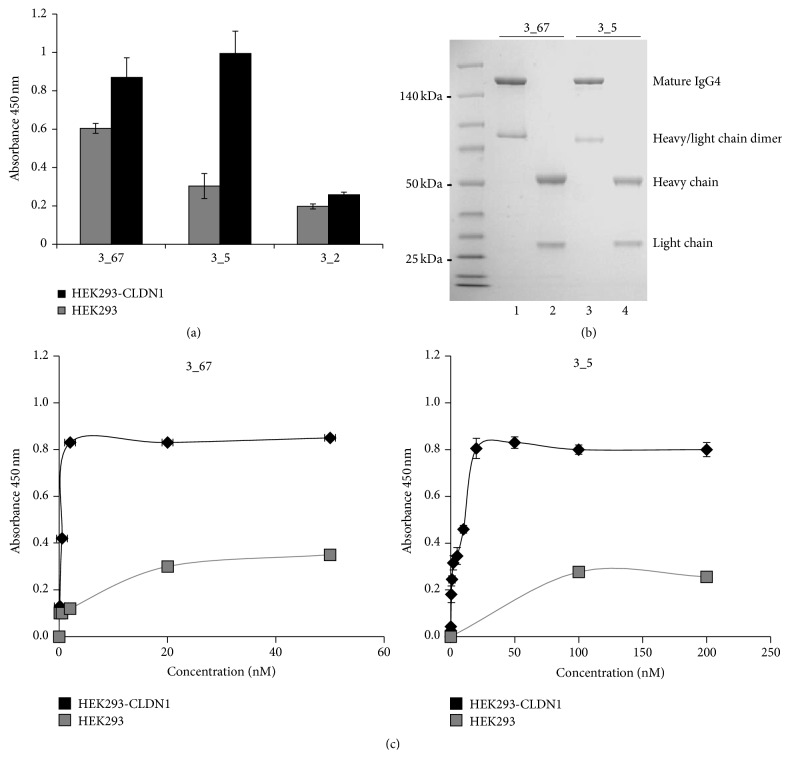
Evaluation of binding for scFv phages and IgG4. (a) The panel shows the binding of scFv phages, clones 3_2, 3_5, and 3_67, to HEK293 cells (gray bars) and to cells transduced with CLDN1 vector (HEK293-CLDN1, black bars). Clone 3_2 was discarded, because of nonspecific binding to HEK293-CLDN1 cells. (b) SDS-PAGE analysis of IgG4, converted from the scFv clones 3_5 and 3_67, as indicated. Samples in lanes 1 and 3 were run under nonreducing conditions, so that the whole IgG4 and the heavy-chain/light chain IgG4 dimers were accordingly visualized. Under reducing conditions, the IgG4 preparations showed the fully denatured light and heavy chains, as indicated. (c) The panels show the binding of the IgG4s, converted from scFv fragments 3_5 and 3_67, to HEK293 (gray lines) and to HEK293-CLDN1 (black lines) cells at increasing antibody concentrations.

Purified phage particles for clones 3_2, 3_5, and 3_67 were generated and tested by cell ELISA to validate their binding. Two out of the three tested clones (3_5 and 3_67) showed a specific binding to CLDN1 expressing cells (Figure 3(a)). Clone 3_2, instead, revealed binding to both cell cultures. Thus, we focused on clones 3_5 and 3_67 for further experiments. They were converted into human IgG4 antibodies.  Figure 3(b) shows that the isolated VH and VL regions of these clones actually generate full antibodies. They were also tested in ELISA to validate their binding to CLDN1 exposed on the surface of HEK-293 cells.  Figure 3(c) shows that the corresponding antibodies actually maintain the ability to bind specifically CLDN1 expressing HEK-293 cells, as for the corresponding scFvs, from which they were generated.

## 4. Discussion

In this paper, we report a complete workflow for HTS-based isolation of scFv phagemid clones binding to native CLDN1, a cell surface protein involved in HCV infection. HTS-based screening of phage display libraries starts to become a useful method to isolate putative scFvs for antigens involved in diseases, ranging from viral infections to cancer. This approach may have some advantages, compared to the classical screening schemes, such as the possibility to comparatively evaluate the complexities of the sublibraries from each selection cycle, and the corresponding enrichments of phage clones, from which to derive functional antibodies against a given antigen. This allows us, for instance, to decide whether to stop, or to continue the screening for a given antigen. In our case, the screening strategy was composed of four selection cycles, each one characterized by progressive decreases in entropy. Cycle 3, however, showed maximal enrichments for most clones, since the majority of the 75 scFv constructs, selected for further analysis, dropped their relative representation during selection cycle 4. The main interpretation for this occurrence is that cycle 4 represents a* plateau* for our selection, thus, rendering ineffective additional selection cycles.

During classical screening procedures, much effort is dedicated to repetitive tests, isolation and sequencing of clones, at completion of multiple, downstream selection cycles. Following a HTS-based screening, instead, each of the enriched clones is known in advance and then tested for binding at a single occurrence. Thus, HTS-based screening will reveal the widest possible set of enriched clones, limiting the possibility to lose good binders during repetitive isolation and characterization of active scFvs. Our experimental setup also provides a sustainable alternative to classical screening, since HTS costs are kept to the lowest, combining multiple samples in a single sequencing run. Accordingly, after having performed 4 cycles of selection, we combined cycles 1 and 4 in a run and cycles 2 and 3 in an additional run. The Illumina MiSeq platform was used, demonstrating its proper adaptability to a screening approach. The versatility and the cheap costs (on average, 1,000 USD per run in the international market) of our approach may expand the applicability of such HTS-based screening to the selection of scFv clones for multiple targets.

There is, however, a disadvantage in the use of HTS-based screening, compared to classical approaches. The latter, in fact, allows for direct isolation of phagemid DNA for biochemical validation of binding, via production of soluble scFv protein fragments. On the contrary, there is the need, once the enriched clones have been identified, to recover them from DNA preparations of the enriched sublibraries. Some methods have been developed, to overcome the problem of recovering selected clones; one of them was based on overlapping PCR reactions. These allowed the reconstruction of full scFvs from 2 PCR products, corresponding to VH and VL [18]. An additional method provides single-step isolation of complete phagemid DNA, via a thermostable DNA polymerase and DNA ligase, using an inverse PCR application with 5′-phosphate oligonucleotides [20, 21]. The latter method is, like the one implemented in this paper, based on single-step recovery. It was shown to be highly effective, allowing recovery of a single scFv clone, spiked into a library, and represented to 0.0025% of the total DNA [21]. It was also effective in the recovery of scFv clones, bearing short HCD3 sequences, due to the design of one oligonucleotide primer at the boundary between HCD3 and FR4 region, and of the second primer within FR4. Our approach was fully validated within an experimental screening for CLDN1 antibodies. We were indeed able to identify 75 potential binders, from which we decided to isolate 3 representative scFv clones, selected according to the length of the corresponding HCDR3 regions (range 10–17 amino acids) and within an experimentally validated range of frequencies (from 8%, down to 0.1% of the population of clones, represented within cycle 3). Its preliminary implementation by Zhang and coworkers [19] was not fully exploited, since these authors focused on hybridization-mediated capture of the selected clones, via hybridization with biotinylated oligonucleotides, designed within HCDR3 regions. Since the identification of an effective mAb against SR-BI [1, 3, 7] our groups are actively isolating novel mAbs against cellular proteins involved in HCV infection [22]; accordingly, a wide search and characterization of novel antibodies, preventing viral entry through the tight-junction protein CLDN1, is in progress. In the present work, two out of the three selected clones showed good binding specificities to CLDN1 expressing cells, 3_5 and 3_67; clone 3-2, however, although highly enriched, did not generate a specific binding. Its positive selection could represent a combination of a biological advantage and of the peculiarity of the system used for screening, consisting of native CLDN1, expressed on the cell surface. The epitope selected by clone 3_2 may represent a very abundant protein expressed on HEK-293 cells, so that the corresponding scFv is not efficiently removed during the negative selections. During a classical screening such clone would have been selected and discarded at each cycle, after repeated testing; the HTS-based screening, instead, allows us to discard it at the first/unique characterization of binding.

The validity of the whole procedure, from screening to antibody production, was verified, since the antibodies generated by scFv conversions of clones 3_5 and 3_67 were rapidly and efficiently obtained; they fully recapitulated the binding properties of the scFv from which they were derived. As a whole, the application of the complete pipeline proposed within this work, characterized by low costs and high effectiveness, may guarantee rapid, sustainable, and successful isolation of antibodies for multiple proteins against native antigens involved in human diseases.

## 5. Conclusions 

In this paper, we screened scFv “phage display” library on suspension cultures of HEK-293 cells expressing CLDN1 on plasma membrane, from which we successfully isolated specific CLDN1 binders. The optimized high-throughput sequencing approach, followed by a single-step recovery of representative, full scFv constructs and their conversion to IgG4 antibodies, demonstrated the versatility and scalability of the procedure, to obtain rapid and cheap isolation of antibodies for virtually any native antigen involved in human diseases.

## Abbreviations

CLDN1: Claudin-1
dsDNA: Double-stranded DNA
HCDR: Heavy-chain complementarity determining region
HTS: High-throughput sequencing
mAb: Monoclonal antibody
scFv: Single-chain fragment variable
VH: Heavy chain variable region
VL: Light chain variable region.
